# Predictive Modeling Analysis for the Quality Indicators of Matsutake Mushrooms in Different Transport Environments

**DOI:** 10.3390/foods12183372

**Published:** 2023-09-08

**Authors:** Yangfeng Wang, Xinyi Jin, Lin Yang, Xiang He, Xiang Wang

**Affiliations:** 1 Beijing Laboratory of Food Quality and Safety, College of Engineering, China Agricultural University, Beijing 100083, China; yangfengwang@cau.edu.cn (Y.W.); xinyijin@cau.edu.cn (X.J.); hexiang@cau.edu.cn (X.H.); 2 College of Food Science, Tibet Agricultural and Animal Husbandry College, Linzhi 860000, China; yanglin@xza.edu.cn

**Keywords:** Matsutake mushroom, cold chain, gas conditioning, food control, quality prediction

## Abstract

Matsutake mushrooms, known for their high value, present challenges due to their seasonal availability, difficulties in harvesting, and short shelf life, making it crucial to extend their post-harvest preservation period. In this study, we developed three quality predictive models of Matsutake mushrooms using three different methods. The quality changes of Matsutake mushrooms were experimentally analyzed under two cases (case A: Temperature control and sealing measures; case B: Alteration of gas composition) with various parameters including the hardness, color, odor, pH, soluble solids content (SSC), and moisture content (MC) collected as indicators of quality changes throughout the storage period. Prediction models for Matsutake mushroom quality were developed using three different methods based on the collected data: multiple linear regression (MLR), support vector regression (SVR), and an artificial neural network (ANN). The comparative results reveal that the ANN outperforms MLR and SVR as the optimal model for predicting Matsutake mushroom quality indicators. To further enhance the ANN model’s performance, optimization techniques such as the Levenberg–Marquardt, Bayesian regularization, and scaled conjugate gradient backpropagation algorithm techniques were employed. The optimized ANN model achieved impressive results, with an R-Square value of 0.988 and an MSE of 0.099 under case A, and an R-Square of 0.981 and an MSE of 0.164 under case B. These findings provide valuable insights for the development of new preservation methods, contributing to the assurance of a high-quality supply of Matsutake mushrooms in the market.

## 1. Introduction

The Matsutake mushroom (*Tricholoma Matsutake*) is an ectomycorrhizal fungus that possesses rich nutritional value and has been found to have immunomodulating and antioxidant properties, having high medicinal value, making it popular among consumers [1,2,3,4]. From January to October 2022, 296.55 tones of Matsutake mushrooms in China were exported, with an export value of 116 million yuan, and the average export price increased to 389.6 yuan/kg, which indicates that Matsutake mushrooms are of high commercial value [5]. However, Matsutake mushrooms have extremely strict requirements for their growing environment. Any insect damage, vandalism, or unsuitable temperature or light during growth can cause irreversible damage [6]. The species of Matsutake mushrooms studied in this paper primarily grow in the forest areas of southwest and northeast China, at a distance of over 20 km from residential regions [7]. These Matsutake mushrooms exhibit high biological activity and maintain high metabolic intensity even after harvesting. Failure to implement appropriate preservation measures can lead to the rapid decomposition of Matsutake mushrooms during transit, within only 1–2 days [8]. Several quality factors undergo degradation in the post-harvest stage, including moisture loss, discoloration, texture alteration, flavor deterioration, and nutrient value reduction, which contribute to a decline in the overall value of the Matsutake mushrooms [9,10]. To ensure that the Matsutake mushrooms retain their highest value, local harvesters have to set off early in the morning and sell them within two hours of descending the mountain. Given the labor-intensive, economically inefficient, and wasteful nature of the process, it is imperative to prioritize the post-harvest preservation of Matsutake mushrooms as a critical step in the overall Matsutake mushroom supply chain.

Currently, the Matsutake mushroom industry uses three main methods for preservation: drying, refrigeration, and modified atmosphere packaging. Drying is considered an effective way of maintaining the stability of food items, but can cause the loss of moisture and nutrients and a poor taste [11,12,13]. Refrigeration has become the most widely used method of preserving Matsutake mushrooms today by mitigating the biological activity of the food, which effectively extends its shelf life [14]. The use of gas-conditioned packaging is a new method of preserving Matsutake mushrooms that has appeared in recent years, which can maximize the freshness and nutritional value of Matsutake mushrooms, but the price is higher [15,16]. Refrigeration and the use of modified atmosphere packaging have great potential as effective methods of extending the shelf life in a market that prefers fresh Matsutake mushrooms.

In the current Matsutake mushroom production areas, only a handful of companies engage in the timely preservation and packaging of the Matsutake mushroom, and complete industry standards and systems have not been established. To promote the development of the Matsutake mushroom industry, one of the crucial elements is the establishment of a quality standards model to ensure the preserved and packaged Matsutake mushrooms meet the required quality standards. In the process of establishing a quality standards model, different models can be built based on experimental data, and the models’ performance can be compared to derive the best predictive model. It is also necessary to optimize the predictive model to achieve better results [17,18,19]. Currently, numerous studies have been carried out to model food storage processes. Wang et al. modeled changes in the quality of tilapia during frozen storage to predict the quality at −40 °C and −8 °C throughout the storage period [20]. Chong et al. developed a model to investigate the effect of stage cutting on the gas separation performance of hollow fiber membrane modules to extend the life of fruits and vegetables during transport and storage [21]. When modeling an indicator, there are several effective and commonly used methods in various industries such as the food, agriculture, forestry, resource, and environmental industries, including multiple linear regression (MLR), support vector regression (SVR), and artificial neural networks (ANNs), as noted in previous studies [22,23,24]. MLR is a suitable choice for target indicators with multiple variable influences, as it allows the weight parameters of the factors to be varied, although it is prone to overfitting when dealing with high-dimensional data, which may affect the robustness and accuracy [25]. SVR is a specialized branch of SVM that obtains the weights of the network by solving a quadratic programming problem with linear constraints, which can effectively solve non-linear regression models and has better results for small batches of data [26]. Li et al. established optimal prediction models for salmon storage times by utilizing support vector regression coupled with variable selection methods, revealing salmon quality evaluation methods under different temperature conditions [27]. For non-linear regression problems with multiple inputs and outputs, the use of an ANN is quite a useful method that can establish relationships between input and output variables through the learning process itself, without the need for human definition [28]. Sampaio et al. employed ANN and MLR models to predict rice quality based on physical grain parameters for qualitative and quantitative analyses [29]. While these methods provide constructive ideas for modelling quality indicators, further research and the selection of appropriate regression methods are necessary to develop predictive models for quality indicators during storage of Matsutake mushrooms.

In the recent research on Matsutake mushrooms, the main focus is on internal traits, growth characteristics, preservation methods, shelf-life prediction, etc. [1,2,10,30]. However, there is currently a lack of relevant research on the modeling of quality indicators for post-harvest storage of Matsutake mushrooms, which forms an urgent gap in the field. Therefore, the aim of this paper is to investigate the quality changes of Matsutake mushrooms in different post-harvest storage environments to provide insights into the development of effective storage strategies for Matsutake mushrooms. MLR, SVR and ANN methods are utilized to provide accurate prediction and control of quality changes in Matsutake mushrooms, mitigating the negative economic impact of suboptimal storage conditions.

## 2. Materials and Methods

### 2.1. Architecture of the Experiment

**Figure 1:**
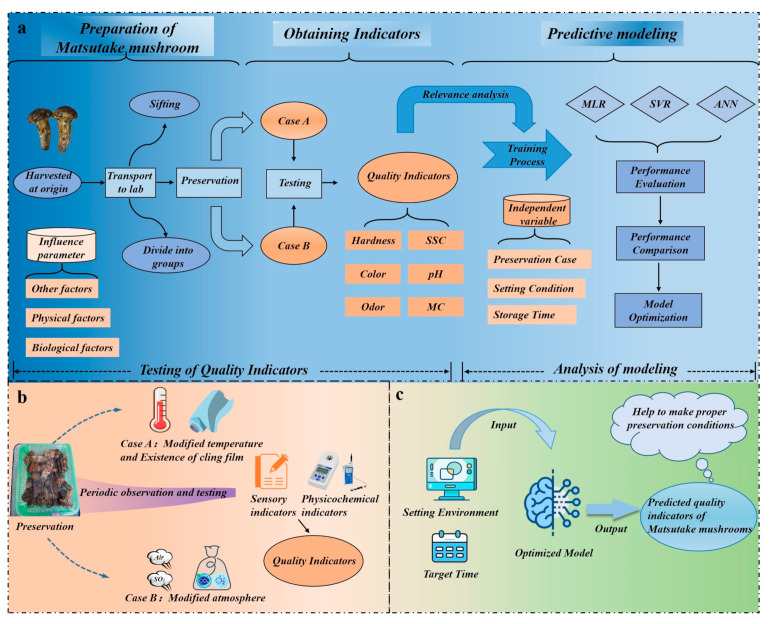
The architecture of the experiment. (**a**) Procedure for conducting the experiment. (**b**) Matsutake mushrooms are treated in two cases. (**c**) Using the model to guide Matsutake mushroom preservation.

The architecture of the entire experiment can be divided into three parts, as depicted in Figure 1a: preparation of the Matsutake mushroom, obtaining indicators, and predictive modeling. Matsutake mushrooms are harvested and transported to the laboratory in Nyingchi for further processing. Figure 1b demonstrates that the laboratory creates two storage cases (Case A, Case B), each with two variables. Throughout the storage period, random samples of Matsutake mushrooms are periodically removed for in-process quality observation. Utilizing a multi-method assessment approach, quality indicators are collected as datasets for model training. The trained model is able to guide Matsutake mushroom preservation, which is shown in Figure 1c.

### 2.2. Materials and Transport Environment Preparation

#### 2.2.1. The Harvesting of Matsutake Mushrooms

The Matsutake mushrooms used in this paper were harvested from the forest area of Nyingchi City, Tibet. Fresh Matsutake mushrooms were collected in the morning of the experiment, cooled to approximately 5 °C in a thermostatically controlled refrigeration unit (Shanghai Yiheng Technology, Shanghai, China), and transported to the Tibetan Institute of Agriculture and Animal Husbandry laboratory at 4 ± 1 °C within 2 h of harvest. A soft brush was used to remove debris from their surfaces in the laboratory to prepare the Matsutake mushrooms for the experiment. Any damaged, deformed, or diseased Matsutake mushrooms were excluded to ensure that the experimental samples were 80–90% mature, fresh, and intact. A total of 246 Matsutake mushrooms were used for this experiment.

#### 2.2.2. Storage Environment Settings

The experiment aimed to investigate the effects of different variables on the quality indicators of Matsutake mushrooms during storage according to previous research [17]. It was divided into two parts, each with its own set of independent variables and experimental settings.

In Case A, the study focused on two independent variables: preservation temperature and cling film sealing. To facilitate experimentation, the Matsutake mushrooms were evenly apportioned into six distinct groups, labeled as $G_{a1}$ to $G_{a6}$, and placed within six polypropylene (PP) skeleton baskets. Out of the six groups, four were selected at random to undergo an additional step involving the complete encapsulation of the skeletonized frames with polyethylene (PE) cling film (Weide New-material Corp., Xuzhou, China). This subset was categorized as “Sealed by cling film”. Conversely, the remaining two groups were designated as the “without sealing” group, because of the non-existence of clingfilm.

Within the “Sealed by cling film” group, a diverse range of preservation temperatures was implemented across four gradient levels: 0 °C, 4 °C, 10 °C, and 20 °C. In contrast, the “without sealing” group was subjected to two distinct temperature gradients: 0 °C and 4 °C. Comprehensive details pertaining to the environmental conditions employed for each group can be found in Table 1.

In Case B, the focal independent variables investigated was related to gas regulation parameters [31]. To establish a controlled experimental setting, a configuration involving multiple plastic bags (Weide New-material Corp., Xuzhou, China) was adopted to create hermetic enclosures for the Matsutake mushrooms. Specifically, each plastic bag accommodated two Matsutake mushrooms. The material of the vacuum preservation bags was polyethylene (PE) with a size of 0.15 × 0.2 m. 

After the Matsutake mushrooms were placed, these plastic bags were closed and vacuumed using a vacuum packaging machine (Ningbo, China). Subsequently, the bags were filled with a standardized gas mixture obtained from Chengsui Oxygen Production Company (Nyingchi, China) through. Comprehensive details regarding the specific parameters of gas composition can be accessed in Table 2.

For consistency, each group of bags was maintained within a sealed environment at a constant temperature of 4 °C, with the composition of the enclosed gas serving as the independent variable. All the sealed bags were stored in a biochemical incubator at a temperature and relative humidity of 4 ± 1 °C and 90% RH.

### 2.3. Quality Indicators Measurement of Matsutake Mushrooms

#### 2.3.1. Quality Evaluation Indicators

To detect changes in the quality of Matsutake mushrooms during storage, this study was conducted using sensory and physicochemical indicators. The evaluation of sensory indicators included hardness (cap and stem), color, and odor of Matsutake mushrooms. The physicochemical indicators consisted of moisture content (MC), pH, and soluble solids content (SSC). For the groups where temperature and sealing were the independent variables, two Matsutake mushrooms were sampled at 24 h intervals for each group. For the groups where the gas environment was the independent variable, one plastic sealed bag containing two Matsutake mushrooms was sampled at 48 h intervals for measurement. 

To ensure the accuracy and reliability of the measurements, it is necessary to follow certain protocols. Firstly, rubber gloves should be worn during all measurements to prevent contamination and ensure safety. Secondly, forceps should be used for sample transfer to minimize the risk of introducing external factors that may affect the measurement results. Finally, it is crucial to maintain a dry and clean environment throughout the measurement process to avoid any interference from external factors that may affect the measurement.

#### 2.3.2. Sensory Indicators

A team of trained evaluators consisting of a local collector, a buyer, a cook, and two consumers were invited to participate in the sensory evaluation. The evaluators rated the sensory indicators of Matsutake mushrooms by observing and filling out forms daily and recorded their scores separately for each criterion. The average value of each evaluator was calculated and recorded as the final data. This study divided the range of sensory evaluation scores into four levels, and the specific evaluation criteria are shown in Table 3.

#### 2.3.3. Physicochemical Indicators

To prepare the Matsutake mushroom samples, each Matsutake mushroom was sliced into 15–18 pieces with a thickness of approximately 1 mm. The moisture content (MC) was measured using a moisture meter (Shenzhen Crown and moisture meter technology Co., LTD., Shenzhen, China).

A sample of 5 g of the prepared Matsutake mushroom was mixed with 20 mL of 0.05 mol/L lactic acid buffer solution (pH 6.8) containing 1% polyvinylpyrrolidone (PVP) and ground uniformly with a mortar and pestle under ice bath conditions. The resulting homogenate was then filtered through four layers of gauze and centrifuged at 10,000–15,000 rpm for 10 min at 0–4 °C. The supernatant was collected to measure pH and solid soluble content (SSC). The pH value was measured using a pHB-4 pH meter (INESA.CC, Shanghai, China). The content of SSC was measured using a glycometer refractometer (LH-B55, Hangzhou, China). 

Three independent experiments were conducted on each Matsutake mushroom sample to determine moisture content (MC), pH, and solid soluble content (SSC). Subsequently, the average of the results of these three different experiments was calculated to create the final datasets.

### 2.4. Quality Prediction Models

After collecting quality indicators of stored Matsutake mushrooms under different conditions, there is a need for accurate and efficient methods to model these indicators. Three different models MLR, SVR, and ANN were selected for this study. Both MLR and SVR are multiple-input single-output regression models, while ANN is a multiple-input multiple-output predictive model. 

In this study, we created training and test sets for MLR and SVR with a split of 70% for training and 30% for test. For the ANN model, we employed a more detailed partitioning, allocating 70% for training, 15% for test, and 15% for validation. Details of the dataset can be seen in Table 4.

#### 2.4.1. Multiple Linear Regression (MLR)

MLR is a useful approach for evaluating target indicators that are impacted by several variables because it allows for the adjustment of the weight parameters of the factors [25]. Given that the quality of Matsutake mushrooms is influenced by multiple factors, including storage temperature and gas environment, simple linear regression (SLR) may not be sufficient to capture the changes in quality indicators since it considers only one predictor variable to estimate the outcome variable [28]. In contrast, MLR is an appropriate regression model used to represent a linear relationship between independent and dependent variables when multiple characteristics are utilized as inputs [32]. The linear form of the model is shown in Equation (1) [33]. The $i_{th}$ dependent variable predicted by the model, which includes hardness, color, odor, pH, SSC, and MC. The $x_{i}$ represents the $i_{th}$ independent variable, which is the storage condition and retention time, respectively. $\beta_{0}$ is the intercept, $\beta_{i}$ is the linear regression coefficient.

(1) $$y=\beta_{0}+\sum_{i=1}^{3}\beta_{i}x_{i}+\varepsilon$$

In this study, interaction (2FI) effects are incorporated using the equation presented in Equation (2), $\beta_{ij}$ is the interaction regression coefficient [34,35]. By including interaction terms, this approach accounts for the non-independence of multiple independent variables and provides a more precise depiction of the relationship between the independent and the dependent variables [36]. This technique allows for a more accurate description of how the effects of different independent variables interact with each other.

(2) $$y=\beta_{0}+\sum_{i=1}^{3}\beta_{i}x_{i}+\sum_{i=1}^{3}\sum_{j=i+1}^{3}\beta_{ij}x_{ij}+\varepsilon$$

#### 2.4.2. Support Vector Regression (SVR)

SVR applies the principles of Support Vector Machines (SVM) to regression problems, which obtain the weights of the model by solving a quadratic programming problem with linear constraints. SVR has a significant advantage in dealing with small batches of data, as the model parameter estimation is formulated as a quadratic optimization problem with the objective of minimizing structural risk [26]. This approach effectively addresses the issue of overfitting and leads to improved generalization of the model [37]. 

**Figure 2:**
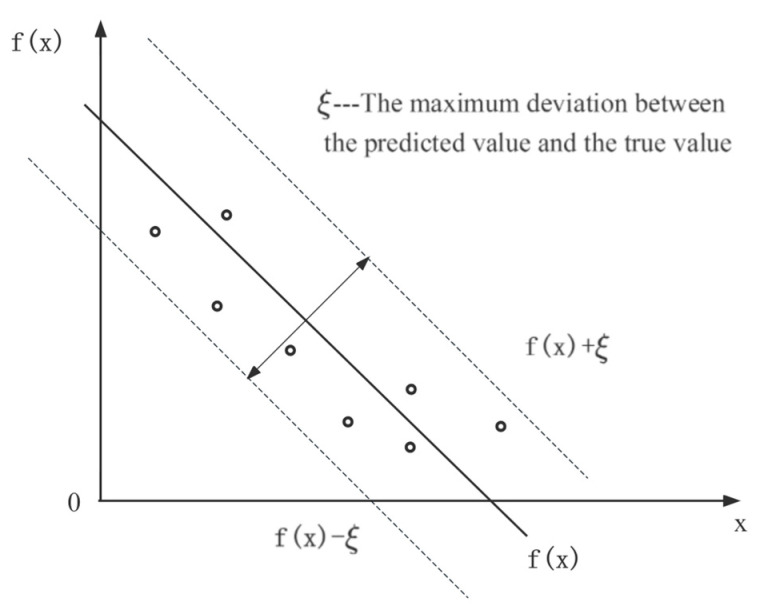
Algorithm principle of SVR.

The algorithm principle of SVR is shown in Figure 2. In this study, by mapping the input data, including storage conditions and retention time, to a high-dimensional feature space and constructing a kernel function, the problem is solved with a linear regression function [38]. SVR incorporates an interval band surrounding the linear function, with a width of ξ (tolerable bias). This interval band is designed to accommodate the variability and uncertainty in the prediction. In SVR, the focus is not solely on individual sample losses that fall within the band, but rather on overall model performance. Therefore, the prediction of SVR for each dependent variable such as hardness, color, odor, pH, SSC, and MC is correct when it falls within an interval band of width ξ [39].

The kernel function selected for SVR plays a pivotal role in determining the mapping of the training set to high-dimensional space and consequently, the overall performance of the SVR model. For this study, the Radial Basis Function (RBF) kernel has been opted, which requires tuning of a unique hyperparameter. The corresponding equation is presented in Equation (3).

(3) $$\kappa \left(x_{i},x_{j}\right)=e^{\left(-\frac{{\left|\left|x_{i},x_{j}\right|\right|}^{2}}{2\sigma^{2}}\right)}\;\in \left(0,1\right]$$

#### 2.4.3. Artificial Neural Network (ANN)

The ANN model is developed through the utilization of the backpropagation technique in conjunction with a feed-forward neural network structure [40]. This approach is particularly useful for non-linear regression problems that involve multiple inputs and outputs. The ANN model comprises a series of connections with unique weights and biases, which interconnect three distinct layers, including the input, hidden, and output layers. A theoretical advantage of the ANN is that it can establish relationships between input and output variables through the learning process itself, without the need for human definition [28].

**Figure 3:**
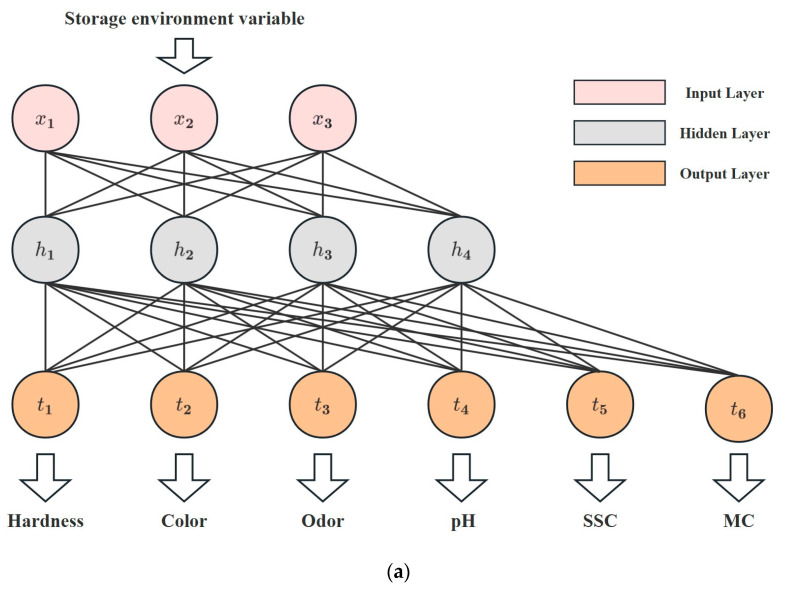

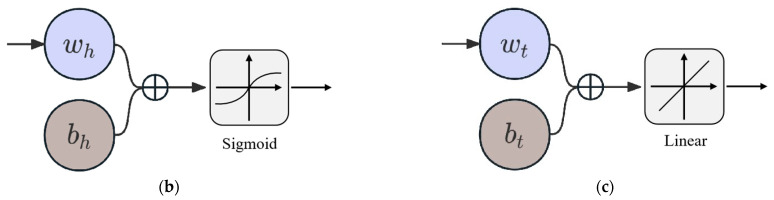
ANN model. (**a**) Structure of the ANN model. (**b**) Neuron in the hidden layer. (**c**) Neuron in the output layer.

This research explores the utilization of an ANN model for evaluating the quality of Matsutake mushrooms during different preservation environments. The structure proposed in this study is shown in Figure 3a and consists of an input layer, a hidden layer, and an output layer. Specifically, the input layer consists of three neurons representing storage conditions and preservation time. As shown in Figure 3b, the hidden layer consists of four neurons, all using Sigmoid as the activation function. Figure 3c demonstrates the linear output neurons. Six linear output neurons make up the output layer, representing hardness, color, odor, pH, SSC, and MC.

### 2.5. Statistical Methods

This study capitalized on specialized software to ensure rigorous analysis and efficient model development. Microsoft Excel 2019 (Microsoft, Redmond, WA, USA) was employed for initial data organization and processing. The Neural Net Fitting and Regression Learner toolboxes in MATLAB 2022a (MathWorks, Natick, MA, USA) facilitated precise model creation and refinement. Additionally, OriginPro2023 (OriginLab, Northampton, MA, USA) facilitated regression analysis and visualization, enhancing result interpretation.

The assessment of our model’s performance was achieved through the utilization of established metrics. The Mean Squared Error (MSE) was employed to gauge predictive accuracy by measuring the disparities between predicted and actual values. Complementing this, the Root Mean Square Error (RMSE) was utilized to standardize error assessment through the computation of the square root of the MSE. Furthermore, we integrated the Mean Absolute Error (MAE) to evaluate the precision of predictions, focusing on the average magnitude of errors, regardless of their direction. In addition to these metrics, the Coefficient of Determination ($R^{2}$) was also incorporated to measure the goodness of fit by quantifying the linear relationship between variables. Formulas (4)–(7) show the specific calculation of these established metrics.

(4) $$MSE=\frac{1}{m}\sum_{i=1}^{m}{\left(y_{i}-{\hat{y}}_{i}\right)}^{2}$$

(5) $$RMSE=\sqrt{\frac{1}{m}\sum_{i=1}^{m}{\left(y_{i}-{\hat{y}}_{i}\right)}^{2}}$$

(6) $$MAE=\frac{1}{n}\sum_{i=1}^{n}\left|{\hat{y}}_{i}-y_{i}\right|$$

(7) $$R^{2}=1-\frac{\sum_{i=1}^{n}{\left(y_{i}-{\hat{y}}_{i}\right)}^{2}}{\sum_{i=1}^{n}{\left(y_{i}-\bar{y_{i}}\right)}^{2}}$$

Furthermore, K-fold Cross Validation was employed during model training to enhance reliability. All statistical assessments, including *RMSE, MAE, MSE,* and $R^{2}$, were executed within MATLAB 2022a, ensuring a comprehensive understanding of the model’s predictive performance.

## 3. Results and Discussion

### 3.1. Relevance Analysis of Matsutake Mushroom Quality Indicators

**Figure 4:**
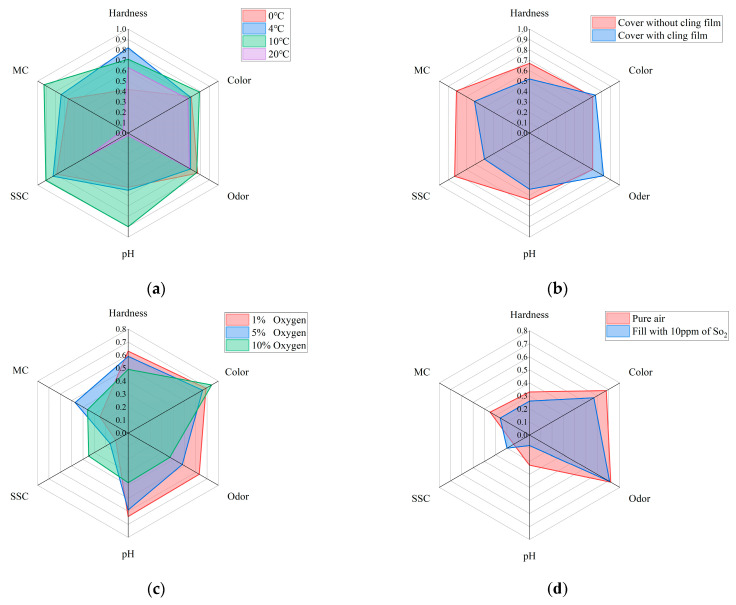
Radar plot of the correlation between quality indicators and storage time of Matsutake mushrooms under different preservation environments (MC stands for moisture content and SCC stands for soluble solids content). (**a**) Under different preservation temperature. (**b**) With/without cling film. (**c**) Under different oxygen concentrations. (**d**) With/without ${\mathrm{SO}}_{2}$ in pure air.

In order to ensure the correct selection of quality assessment parameters and to improve the accuracy of the analysis of changes in the quality of Matsutake mushrooms in different storage environments, this study employed an independent analysis of all samples to establish a time-quality correlation. The results of the study are visually represented in a radar chart as shown in Figure 4. In this representation, the correlation coefficients between time and the respective variables served as coordinates along the radial axes. The radar plots effectively demonstrated distinct trends in the quality indicators of Matsutake mushrooms across varying storage conditions. These six quality indicators exhibited strong correlations with time. Hence, they are deemed suitable as reliable quality indicators for assessing the overall quality of Matsutake mushrooms.

### 3.2. Performance Analysis of Established Models under Two Environmental Conditions

In this study, the datasets are divided into two different groups based on the environmental conditions under which the data are collected. The first group, referred to as Case A, involves the modification of temperature and the use of cling film as factors influencing the storage environment. In Case A, the correlation coefficients of MC and pH decrease substantially at 20 °C, but the correlation coefficients of all six quality indicators are higher in general, which can reflect that each quality indicator has a strong correlation with the storage time. Therefore, the prediction models built from the six quality indicators are meaningful. For the two single output models, MLR and SVR, the performance of the six models built for the six quality indicators is analyzed separately to obtain the best model performance in predicting which quality indicator.

The second group, referred to as Case B, focuses on the effect of the modified atmosphere on storage conditions. In Case B, the whole correlation coefficient between MC and SSC is lower than 0.5, while the correlation coefficients of the remaining four quality indicators are higher in general. The correlation of MC and SSC with time is therefore poor. It indicates that the prediction models established in the four aspects of hardness, color, odor, and pH are more reliable and meaningful. For MLR and SVR, the focus is on analyzing the performance of the models built for the four quality indicators to obtain the best model performance in predicting which quality indicator.

#### 3.2.1. Performance Analysis of MLR

The performance of the built MLR model is presented in Table 5. For the environment of Case A, the performance of the MLR model built for six quality metrics is analyzed, in which MLR shows the best performance in SSC, the R-Square reaches 0.760, RMSE reaches 0.248, and MAE reaches 0.188, indicating a high degree of accuracy. Additionally, the performance of the MLR model built for the four quality indicators with high correlation coefficients is analyzed in Case B, in which MLR shows the best performance in predicting color with an R-Square of 0.42 and an RMSE of 0.669. Overall, the results suggest that the MLR model is more effective in predicting the quality indicators under Case A.

#### 3.2.2. Performance Analysis of SVR

The performance of the built SVR model is presented in Table 6. For the environment of modified sealing and temperature conditions, we analyze the performance of SVR models built for six quality indicators, in which SVR performs best in SSC, the R-Square reaches 0.710, RMSE reaches 0.272, and MAE reaches 0.176. For the environment of modified atmosphere, an analysis of the performance of SVR models built for four quality metrics with high correlation coefficients is conducted, in which SVR shows the best performance in color, the R-Square reaches 0.37, RMSE reaches 0.692, and MAE reaches 0.550. The results suggest that the SVR model is more effective in predicting the quality indicators under modified sealing and temperature conditions. 

#### 3.2.3. Establishment and Performance Analysis of ANN

The performance parameters of the built ANN model are presented in Table 7. As a multi-input and multi-output model, the ANN demonstrates an effective predictive capability for quality indicators. The R-Square values obtained for the test datasets under modified sealing and temperature conditions and modified atmosphere are 0.984 and 0.978, respectively, indicating a high degree of accuracy. Additionally, the MSE values obtained are 0.129 and 0.190, respectively. The performance parameters obtained for the training, validation, and test datasets are all satisfactory. When comparing the performance of the ANN model under different environmental conditions, it is found that the ANN model shows better results and smaller errors under modified sealing and temperature conditions. However, the differences in performance between the two environmental conditions are not significant. Overall, the results suggest that the ANN model is a promising approach for predicting quality indicators of Matsutake mushrooms under different environment conditions.

**Figure 5:**
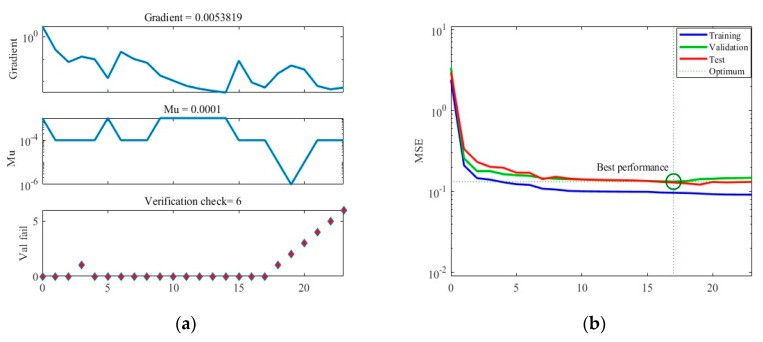

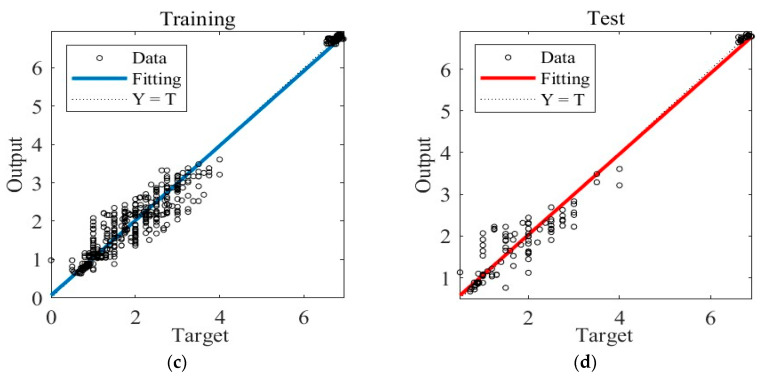
The training process of ANN model under Case A. (**a**) Gradient, Mu, and validation checks during training process. (**b**) Changes in MSE during the training process. (**c**) Predictions of the final ANN on the training datasets. (**d**) Predictions of the final ANN on the test datasets.

Figure 5 illustrates the training results obtained under Case A. In Figure 5a, the graph showcases the gradient descent, changes in Mu (learning rate), and validation checks conducted throughout the training process. The training concludes when six consecutive validation checks show no further reduction in error, resulting in the generation of the final ANN. Figure 5b presents the Mean Squared Error (MSE) loss during training, indicating that the minimum MSE value is achieved in the 17th round of training, after which the loss stabilizes. Figure 5c showcases the ANN’s predictions within the training dataset, while Figure 5d displays its predictions within the separate test dataset.

**Figure 6:**
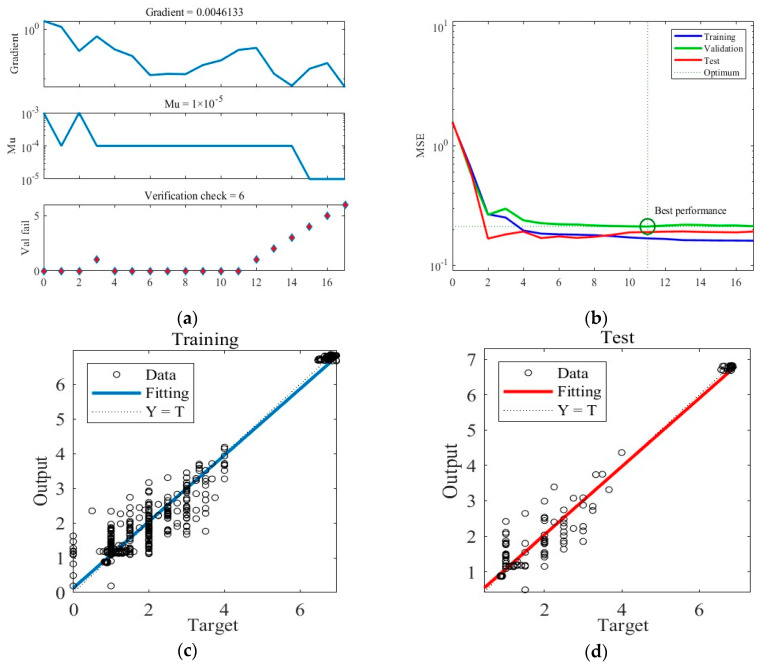
The training process of the ANN model under Case B. (**a**) Gradient, Mu, and validation checks during the training process. (**b**) Changes in MSE during the training process. (**c**) Predictions of the final ANN on the training datasets. (**d**) Predictions of the final ANN on the test datasets.

Similarly, Figure 6 shows the training results obtained under Case B. The training ceasing and the final ANN network outputting after six consecutive validation checks display no reduction in error. The MSE loss during training achieves the minimum in the 11th round and stability thereafter. Figure 6c,d present the predictions of the final trained ANN in the training and test datasets under Case B.

### 3.3. Comparison of the Three Models

#### 3.3.1. Comparison of the Effectiveness of the Predictive Models

**Figure 7:**
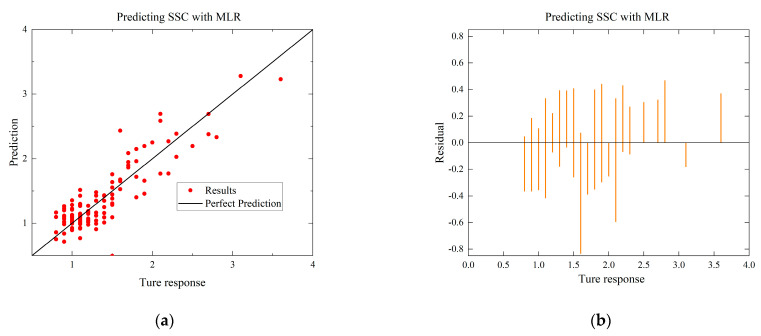

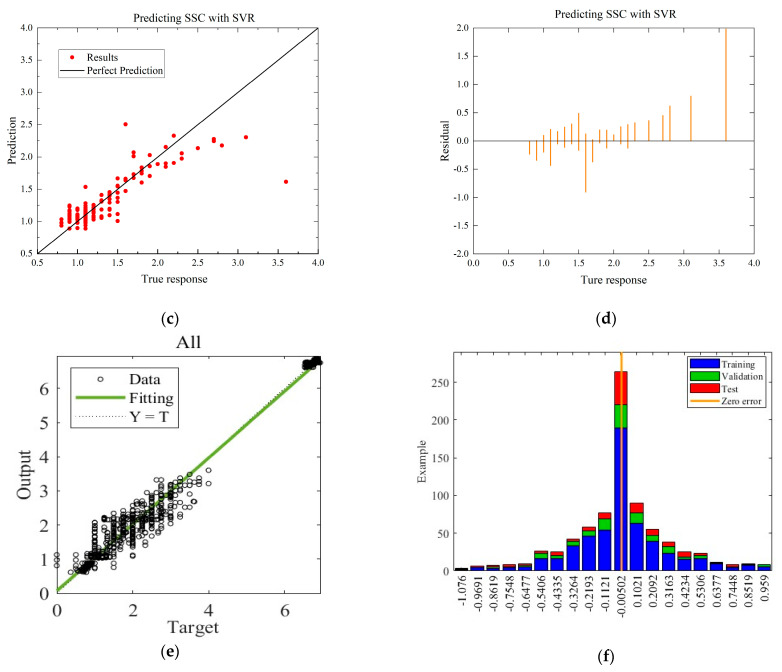
Comparison of regressions under Case A between MLR, SVR, and ANN models. (**a**) Actual-forecast chart of MLR as a representative graph. (**b**) Residual plot of MLR as a representative graph. (**c**) Actual-forecast chart of SVR as a representative graph. (**d**) Residual plot of SVR as a representative graph. (**e**) Actual-forecast chart of ANN. (**f**) Error distribution histogram of ANN.

**Figure 8:**
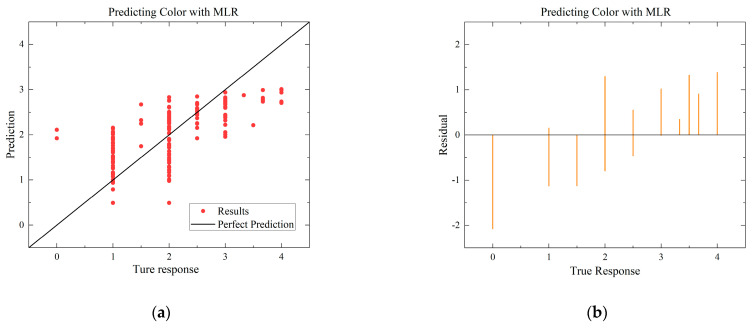

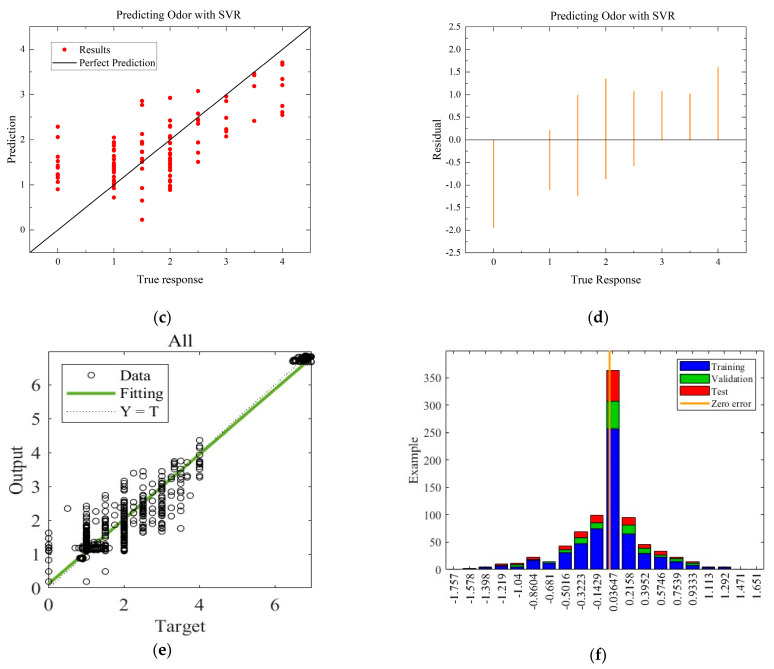
Comparison of regressions under Case B between MLR, SVR, and ANN models. (**a**) Actual-forecast chart of MLR as a representative graph. (**b**) Residual plot of MLR as a representative graph. (**c**) Actual-forecast chart of SVR as a representative graph. (**d**) Residual plot of MLR as a representative graph. (**e**) Actual-forecast chart of ANN. (**f**) Error distribution histogram of ANN.

To compare with the ANN model, the MLR and SVR models with the highest R-squared values for each environmental condition are selected. The residual and predictive regression plots for the MLR and SVR models are presented in Figure 7 and Figure 8, while the error distribution histograms and predictive regression plots are shown for the ANN model. 

When comparing the performance of the models in scatter plots and residual plots, it is found that although the SVR model predicts most distributions closer to the true values than the MLR, a few points deviate significantly, which may be due to noise in the dataset. The ANN model exhibits outstanding performance, with its predicted distributions being the closest to the true values. Additionally, the error distribution histogram indicates that errors are mainly concentrated in the [−0.2, 0.2] interval. This may be attributed to the fact that the ANN model is a multiple-input, multiple-output model, which has a larger training dataset compared to the MLR and SVR models. ANN is able to automatically capture more features and better predict multiple quality metrics with a larger training dataset. Furthermore, it is noted that none of the modeling approaches under Case B exhibited performance as strong as those under Case A. This difference in performance can be attributed to the subtler effects of altering the gas environment on the Matsutake mushrooms. The intricate interplay between the gas composition and the quality indicators of Matsutake mushrooms may pose additional challenges for modeling and prediction. Overall, the results indicate that the ANN model outperforms MLR and SVR in terms of prediction accuracy, particularly when multiple quality metrics are considered. The larger training dataset and the model’s ability to capture complex relationships contribute to its superior performance. Additionally, the variation in performance between Case A and Case B suggests the need for further exploration and understanding of the effects of different storage environments on Matsutake mushroom quality.

#### 3.3.2. Comparison of the Performance Parameters of the Predictive Models

**Figure 9:**
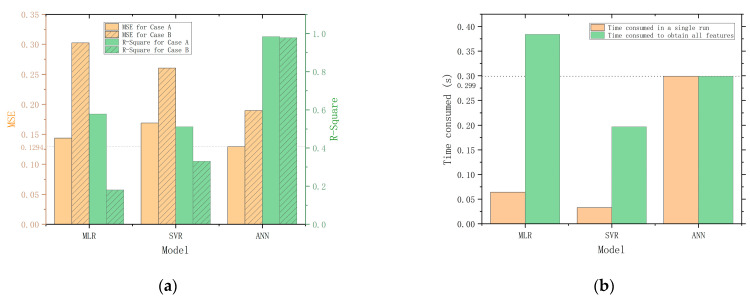
Performance parameters comparison. (**a**) Comparison of MSE and R-Square for MLR, SVR, and ANN. (**b**) Comparison of the time consumed by running MLR, SVR and ANN.

Figure 9 compares the main performance evaluation parameters of MLR, SVR, and ANN. Figure 9a compares the MSE and R-Square of each model. The MSE and R-Square of the MLR and SVR are the mean of the models corresponding to all predictor variables. The result shows that MLR gives better results in Case A, but SVR gives better results in Case B. The ANN model exhibits superior performance compared to the MLR and SVR models. This may be attributed to the increased dimensionality and abundance of information in the three-input, six-output datasets used for training the ANN model, in contrast to the lower-dimensional three-input, one-output datasets used for training the MLR and SVR models. The ability to handle complex and high-dimensional datasets of ANN allows for the extraction of more meaningful patterns and relationships in the data, resulting in more accurate predictions of the quality indicators.

To evaluate the real-time performance of the models, we conduct tests on the elapsed times of each model. The tests are conducted five times on the same PC, and the elapsed times are measured using the program timer of MATLAB. The average of the five tests is calculated and used as the final value for each model. The results of the tests are presented in Figure 9b for comparison. For a single run, the SVR is the fastest, while the ANN is the slowest, taking 9.1 times longer than the SVR and 4.7 times longer than the MLR. However, compared to MLR and SVR, the advantage of ANN is its ability to output all six quality indicators simultaneously, which significantly improves overall efficiency. When taking overall efficiency into consideration, the performance gap between ANN and SVR is reduced to 0.1 s and even surpasses the MLR model. Therefore, the quality prediction model constructed with ANN is the most efficient and accurate in predicting the quality of Matsutake mushrooms under the two storage conditions.

By employing ANN, the quality of Matsutake mushrooms when transported under different storage conditions can be efficiently and accurately predicted and suggestions can be given for proposing new preservation methods. The most appropriate storage and preservation method can be selected according to the prediction results of the model, thus reducing the wastage of Matsutake mushrooms during transport and enhancing their economic value.

To provide a comprehensive understanding of the fitting results achieved by MLR, SVR, and ANN for Matsutake quality indices, we conducted a comparative analysis of their R-Square values in relation to prior research findings, as summarized in Table 8. We selected the optimal outcomes obtained from MLR, SVR, and ANN as representatives for this comparison. Upon reviewing the table, it becomes apparent that the performance of both MLR and SVR in this study falls short of achieving excellence. Consequently, their practical utility for predicting Matsutake mushroom quality indicators appears to be a formidable challenge, a finding consistent with related studies. In contrast, the ANN model consistently exhibits results that align with previous research, showcasing optimal performance and holding promise for further exploration and application. It is noteworthy that, on the whole, both the MLR and SVR models tend to yield lower R-Square values when compared to ANN. This observation underscores the remarkable efficacy of ANN as an intelligent model in the prediction of quality indicators.

#### 3.3.3. Model Optimization

After comparing the predictive effectiveness of three models (MLR, SVR, and ANN) for assessing the quality of Matsutake mushrooms, it is determined that ANN outperforms the other models. To achieve optimal training results, this study employs the Levenberg–Marquardt, Bayesian regularization, and Conjugated Gradient methods for optimizing the ANN model. The results of the training are presented in Table 9 and Table 10, where the training rounds, R-Square, and MSE are provided as metrics for comparing different training outcomes, representing the time consumed, fitting ability, and predictive error, respectively.

In this study, the ANN model is effectively trained for both Case A and Case B. The results reveal consistent trends in the effects observed across different training methods. Specifically, the Levenberg–Marquardt optimization demonstrates the fastest training speed and the fewest training rounds while updating weight and bias values. Bayesian regularization, on the other hand, updates the weight and bias values using Levenberg–Marquardt optimization, minimizing a combination of squared errors and weights to produce a well-generalizing network. However, this method requires the longest training time due to its larger number of rounds. Scaled conjugate gradient backpropagation, which employs gradient calculations instead of Jacobian calculations used by Levenberg–Marquardt or Bayesian regularization, proved to be more memory efficient and thus more suitable for large-scale fitting calculations.

Upon comparing the training results of the three methods, it is observed that Bayesian regularization achieves superior results and demonstrates better generalization ability in predicting Matsutake mushroom quality indicators. Under Case A, the R-Square and MSE values in the test datasets reach 0.988 and 0.099, respectively. Similarly, under Case B, the R-Square and MSE values are determined to be 0.981 and 0.164. Although Bayesian regularization requires a longer training time, such consumption is considered worthwhile due to the results achieved. Therefore, Bayesian regularization is recommended as the preferred method for this study.

The parameters of the ANN model are reported in Table 11 and Table 12, which include the weights and biases of the hidden and output layers. Each neuron is intricately connected to all neurons in the previous layer through a series of weight parameters. These parameters are iteratively refined during the training process, allowing the model to distil salient features from the input environmental variables and make accurate predictions of various quality metrics of Matsutake mushrooms. The model building and optimization strategies are used to make effective suggestions for quality control during the storage of Matsutake mushrooms.

### 3.4. Suggestions for Future Research

In this study, predictive modeling analysis of Matsutake mushroom quality indicators was completed using various models with temperature and sealing conditions and gas conditions as independent variables. It provides feasible recommendations on how to determine the optimal storage conditions for Matsutake mushrooms. However, owing to the limitations inherent, we believe that future research can explore several directions for further advancement. 

Firstly, exploring the most efficient and economical way to accomplish the preservation of Matsutake mushrooms is an important issue in the industry, and incorporating quantitative economic data will provide practical insights for industry stakeholders. Additionally, expanding the scope of the study to include environmental variables such as humidity and atmospheric pressure will enhance the adaptability of the study. Analyzing the interactions between these factors and preservation techniques could result in a more comprehensive approach suitable for different regions and climates. Finally, an important complementary endeavor would be to assess the ecological impacts of modified air-conditioned packaging and other strategies. By assessing their environmental impacts and comparing them to alternatives, this research could guide the industry in making environmentally friendly preservation choices.

## 4. Conclusions

In this study, MLR, SVR, and ANN are employed to predict the changes in Matsutake mushroom quality indicators over time in different storage environments. The results of the comparison demonstrate that ANN exhibits optimal performance, displaying the best fit and the smallest error. Consequently, ANN is determined as the most suitable model. To further enhance the performance of the ANN, the model is optimized using the Levenberg–Marquardt, Bayesian regularization, and Scaled Conjugate Gradient Backpropagation algorithms.

The optimized ANN yields remarkable results, achieving an R-Square of 0.988 and an MSE of 0.099 under Case A, and an R-Square of 0.981 and an MSE of 0.164 under Case B. These findings suggest that ANN outperforms MLR and SVR in predicting quality indicators of Matsutake mushrooms. The outcomes of this study have significant implications for optimizing existing storage methods to maintain optimal Matsutake mushroom quality and taste during storage. Consequently, this improvement will enhance the market competitiveness and add value for Matsutake mushrooms. Furthermore, this study can contribute to the establishment of industry standards and specifications governing the storage and transportation processes of Matsutake mushrooms. These standards will foster improved industry management and standardization, benefiting the overall Matsutake mushroom industry.

## Figures and Tables

**Table 1:** Temperature and sealing method settings in Case A.

Group Number	Temperature	Sealing Method
$G_{a1}$	4 °C	Without sealing
$G_{a2}$	10 °C	Without sealing
$G_{a3}$	0 °C	Sealed by cling film
$G_{a4}$	4 °C	Sealed by cling film
$G_{a5}$	10 °C	Sealed by cling film
$G_{a6}$	20 °C	Sealed by cling film

**Table 2:** Gas condition settings in Case B.

$Group\;Number_{\;}$	Temperature	$O_{2}$	$CO_{2}$	$N_{2}$	Air	$SO_{2}{}_{\;}$
$G_{b1}$	4 °C	1%	21%	78%	-	-
$G_{b2}$	4 °C	5%	17%	78%	-	-
$G_{b3}$	4 °C	10%	12%	78%	-	-
$G_{b4}$	4 °C	-	-	-	100%	-
$G_{b5}$	4 °C	-	-	-	Coexist with ${\mathrm{SO}}_{2}{}_{\;}$	10 ppm

**Table 3:** Scoring standard of Matsutake sensory indicators.

Level	Score	Hardness	Color	Odor
1	4	Good cap elasticity and firm stipe.	Fresh cap with whitish color, milky white stipe, and no browning.	Intense mushroom aroma.
2	3	Relatively good cap elasticity and relatively firm stipe.	Normal color, slight browning.	Normal, no decay odor.
3	2	The cap and stipe begin to soften.	Moderate browning.	Slightly decay odor.
4	1	The cap and stipe begin to soften severely.	Severe browning, mildew appearing.	Foul decay odor.

**Table 4:** Size of the dataset for each model.

Model		Size of Dataset			
		Total	Train	Test	Validation
MLR	Case A	132	92	40	-
	Case B	144	100	44	-
SVR	Case A	132	92	40	-
	Case B	144	100	44	-
ANN	Case A	132	92	20	20
	Case B	144	100	22	22

**Table 5:** Performance of MLR.

Quality Indicators	Environment Changes	RMSE	R-Square	MSE	MAE
Hardness	Case A	0.499	0.390	0.255	0.402
	Case B	0.726	0.260	0.527	0.591
Color	Case A	0.493	0.540	0.244	0.404
	Case B	0.669	0.420	0.447	0.555
Odor	Case A	0.549	0.520	0.302	0.432
	Case B	0.873	0.290	0.761	0.723
pH	Case A	0.065	0.540	0.004	0.045
	Case B	0.083	0.250	0.007	0.064
SSC	Case A	0.248	0.760	0.062	0.188
	Case B	0.198	-0.100	0.039	0.162
MC	Case A	0.048	0.720	0.002	0.029
	Case B	0.015	0.010	0.000	0.012

**Table 6:** Performance of SVR.

Quality Indicators	Environment Changes	RMSE	R-Square	MSE	MAE
Hardness	Case A	0.524	0.320	0.274	0.407
	Case B	0.696	0.320	0.484	0.526
Color	Case A	0.524	0.480	0.274	0.404
	Case B	0.692	0.370	0.479	0.550
Odor	Case A	0.619	0.390	0.383	0.490
	Case B	0.750	0.470	0.563	0.570
pH	Case A	0.066	0.600	0.004	0.043
	Case B	0.076	0.370	0.006	0.054
SSC	Case A	0.272	0.710	0.074	0.176
	Case B	0.170	0.190	0.029	0.138
MC	Case A	0.059	0.570	0.004	0.032
	Case B	0.013	0.250	0.000	0.010

**Table 7:** Performance of ANN.

Process	Case A		Case B	
	MSE	R-Square	MSE	R-Square
Training	0.097	0.988	0.168	0.981
Validation	0.133	0.984	0.211	0.976
Test	0.129	0.984	0.190	0.978

**Table 8:** Comparison of model performance.

Model	Target	R-Square	Ref.
MLR	Matsutake mushroom	0.760	Our work
MLR	Thermal efficiency	0.611	[32]
MLR	Bitter Taste of Peptides	0.892	[41]
GA-MLR	Stability constants	0.915	[42]
SVR	Matsutake mushroom	0.710	Our work
GA-SVR	Stability constants	0.926	[42]
SVM	Bitter Taste of Peptides	0.899	[41]
BPNN	Thermal efficiency	0.905	[32]
ANN	Matsutake mushroom	0.984	Our work
ANN	Yield of paddy	0.990	[43]
ANN	Bitter Taste of Peptides	0.907	[41]
GA-ANN	Stability constants	0.982	[42]

**Table 9:** Evaluation indicators with different training algorithms under Case A.

Training Algorithm	Rounds	Training		Validation		Test	
		R-Square	MSE	R-Square	MSE	R-Square	MSE
Levenberg–Marquardt	17	0.988	0.0967	0.984	0.133	0.984	0.129
Bayesian Regularization	432	0.987	0.103	-	-	0.988	0.099
Scaled Conjugate Gradient Backpropagation	37	0.985	0.125	0.984	0.146	0.986	0.115

**Table 10:** Evaluation indicators with different training algorithms under Case B.

Training Algorithm	Rounds	Training		Validation		Test	
		R-Square	MSE	R-Square	MSE	R-Square	MSE
Levenberg–Marquardt	11	0.981	0.168	0.976	0.211	0.978	0.190
Bayesian Regularization	154	0.980	0.172	-	-	0.981	0.164
Scaled Conjugate Gradient Backpropagation	15	0.977	0.194	0.975	0.225	0.975	0.216

**Table 11:** Optimized ANN network parameters under Case A.

Layer	Weight	Bias
Hidden Layer	$\left[\begin{array}{ccc} 1.306127242 & 0.192355996 & 0.794442514\\ 0.818185115 & -0.52570183 & -1.560114302\\ -0.088281918 & -0.037236378 & -3.105044355\\ 0.38934753 & -2.095714671 & 1.404998291 \end{array}\right]$	$\left[\begin{array}{c} -2.596632578\\ 0.02680374\\ -3.657694846\\ 5.14378194 \end{array}\right]$
Output Layer	$\left[\begin{array}{cccc} 0.798809418 & 0.240278516 & 1.966531145 & 2.05055072\\ -0.646696148 & 0.499620563 & 1.530571548 & -1.218810374\\ -1.614900515 & 0.112634113 & 1.640862849 & 1.089443748\\ 0.385523516 & 0.618507442 & 0.540555042 & 0.88399579\\ -0.52864933 & -0.63262364 & -0.104428257 & -0.654708846\\ 0.696930108 & 0.502350964 & 0.257086288 & 0.527021286 \end{array}\right]$	$\left[\begin{array}{c} 0.202963025\\ 1.449761954\\ -1.222280155\\ -0.289881573\\ -0.169007687\\ 0.73128475 \end{array}\right]$

**Table 12:** Optimized ANN network parameters under Case B.

Layer	Weight	Bias
Hidden Layer	$\left[\begin{array}{ccc} 0.018132334 & 0.165464774 & 3.267012824\\ -3.118422028 & -0.57112634 & 4.843260114\\ 1.562389272 & 0.804846153 & 1.26839667\\ -1.752817151 & -1.263968562 & -2.330988469 \end{array}\right]$	$\left[\begin{array}{c} 3.107971393\\ 1.443593492\\ 1.837455657\\ -2.850637052 \end{array}\right]$
Output Layer	$\left[\begin{array}{cccc} -0.853548538 & 0.043897023 & 0.621332773 & 0.511832669\\ -0.84423459 & -0.167476408 & 0.235504664 & 0.199996017\\ -0.70350823 & -0.209303821 & -0.59166696 & -0.357556521\\ -0.356850808 & -0.245642955 & 0.400708931 & 0.393789284\\ -0.070346719 & 0.208253552 & -1.194834521 & -1.280184584\\ -0.005448051 & -0.167839898 & 0.928780307 & 0.954185546 \end{array}\right]$	$\left[\begin{array}{c} 0.478005011\\ 0.306570825\\ 0.525624559\\ 0.268015927\\ -0.634634887\\ 0.700847775 \end{array}\right]$

## Data Availability

The datasets generated for this study are available on request to the corresponding author.
